# Stabilization and Validation of 3D Object Position Using Multimodal Sensor Fusion and Semantic Segmentation

**DOI:** 10.3390/s20041110

**Published:** 2020-02-18

**Authors:** Mircea Paul Muresan, Ion Giosan, Sergiu Nedevschi

**Affiliations:** Computer Science Department, Technical University of Cluj-Napoca, 28 Memorandumului Street, 400114 Cluj Napoca, Romania; ion.giosan@cs.utcluj.ro (I.G.); Sergiu.Nedevschi@cs.utcluj.ro (S.N.)

**Keywords:** data association, multi-object tracking, sensor fusion, motion compensation, neural networks

## Abstract

The stabilization and validation process of the measured position of objects is an important step for high-level perception functions and for the correct processing of sensory data. The goal of this process is to detect and handle inconsistencies between different sensor measurements, which result from the perception system. The aggregation of the detections from different sensors consists in the combination of the sensorial data in one common reference frame for each identified object, leading to the creation of a super-sensor. The result of the data aggregation may end up with errors such as false detections, misplaced object cuboids or an incorrect number of objects in the scene. The stabilization and validation process is focused on mitigating these problems. The current paper proposes four contributions for solving the stabilization and validation task, for autonomous vehicles, using the following sensors: trifocal camera, fisheye camera, long-range RADAR (Radio detection and ranging), and 4-layer and 16-layer LIDARs (Light Detection and Ranging). We propose two original data association methods used in the sensor fusion and tracking processes. The first data association algorithm is created for tracking LIDAR objects and combines multiple appearance and motion features in order to exploit the available information for road objects. The second novel data association algorithm is designed for trifocal camera objects and has the objective of finding measurement correspondences to sensor fused objects such that the super-sensor data are enriched by adding the semantic class information. The implemented trifocal object association solution uses a novel polar association scheme combined with a decision tree to find the best hypothesis–measurement correlations. Another contribution we propose for stabilizing object position and unpredictable behavior of road objects, provided by multiple types of complementary sensors, is the use of a fusion approach based on the Unscented Kalman Filter and a single-layer perceptron. The last novel contribution is related to the validation of the 3D object position, which is solved using a fuzzy logic technique combined with a semantic segmentation image. The proposed algorithms have a real-time performance, achieving a cumulative running time of 90 ms, and have been evaluated using ground truth data extracted from a high-precision GPS (global positioning system) with 2 cm accuracy, obtaining an average error of 0.8 m.

## 1. Introduction

Dynamic environment perception is an important and demanding topic in the field of autonomous driving and driving assistance systems. Reliably detecting all the traffic participants in the traffic area represents an important aspect in many self-driving car components such as self-localization, collision avoidance, classification and path planning. The challenge of developing reliable systems comes from the complex nature of the real world and the unpredictability that each road user can have. The vehicle has to cope with various cases where its surroundings can be crowded with multiple types of static and dynamic obstacles such as cars, poles, pedestrians, vegetation, bicycles and others. Various types of sensors and measurement techniques are used for solving the complex problem of perception and for meeting all requirements in different complex driving scenarios.

The term modality is used to describe each such acquisition framework. Due to the rich nature of the environmental processes under adverse conditions, a single acquisition method cannot provide a complete understanding of the surroundings. Sensor data fusion is the process of combining incomplete and imperfect pieces of mutually complementary sensory information such that a better understanding of the real world is achieved. The inclusion of multiple datasets that redundantly analyze the same information can offer a more robust measurement; however, it raises questions beyond those related to individual interpretation of each datum. In particular, the deployment of these sensorial systems can lead to problems regarding the correct data association, efficient fusion of information in various weather and working conditions or false detections, among others.

In the case of autonomous vehicles, for gathering 3D data, the perception system can receive information from mono-cameras, stereo cameras, different 3D-LIDARs and RADAR systems. Approaches that use camera systems have attracted attention due to the rich visual information these sensors can provide. Such approaches are compelling because the object class can also be retrieved. Solutions based on mono-cameras are more affordable; however, they are unable to accurately detect the distance to objects when the road is not flat [1]. Stereo camera devices can solve this problem; however, a major limitation of such systems is that they cannot reconstruct the environment reliably for multiple reasons such as bad illumination conditions, solar flares, perspective warping effect and lack of texture, among others.

3D data can also be extracted from the environment using LIDAR sensors. LIDARs use a laser beam to determine the distance between the sensor and a nearby object. Most LIDARs use light with a wavelength of around 900 nm, although there are some variations that use longer wavelengths for obtaining a slightly better performance in rain or fog. By using a rotating mirror, the LIDAR propagates the laser beams across its field of view. The laser pulses are reflected by objects, and these reflections help create a point cloud for each item in the scene. There are multiple types of laser scanners, each with its own advantages and disadvantages.

For example, a LIDAR with only four sweeping rays is useful due to its ability to work at large distances. The drawback of 4 L LIDARs is that they have a small horizontal scanning angle and they provide point clouds with fewer numbers of points. On the other hand, 16 L and 32 L LIDARs can provide multiple 3D points but in a shorter range. LIDAR has a much higher spatial resolution than RADAR, because of the more focused laser beam, the larger number of scan layers in the vertical direction, and the high density of LIDAR points per layer. In general, the problem with LIDAR sensors is that they fail to accurately detect the correct distances to objects in adverse weather conditions such as rain, snow or fog and they cannot compute object speeds directly.

RADARs have been extensively used in the automotive field for many types of applications such as adaptive cruise control, collision warning, collision avoidance [2] or blind spot warning [3]. While other sensors measure velocity by calculating the difference between two readings, the RADAR uses the Doppler effect to measure speed directly. This effect measures the change in frequency of the RADAR waves based on whether the object is moving away from the measurement source or towards it. The Doppler effect is important for sensor fusion because it provides a means to obtain the velocity as an independent parameter and make the fusion algorithms converge much faster. An advantage of RADARs over other sensors is that they can provide measurements to objects without direct line of sight, due to the ability of electromagnetic waves to bounce off hard surfaces. Furthermore, RADARs can spot occluded objects or buildings due to their capacity to see underneath other vehicles. In the automotive industry, RADAR sensors are a viable option because they are the sensors least affected by rain or fog, and have a wide field of view (around 150 degrees) and a long working range of more than 200 m. Due to their physical characteristics, RADARs are easier to integrate into vehicles than LIDARs and do not require constant cleaning, as LIDARs do. RADAR sensors are better at computing the position and velocity of metallic objects in bad weather conditions; however, they fail to detect objects made up of porous plastic or wood. A big disadvantage of RADAR systems is that they discard objects in order to avoid over-reporting. Even though this feature may be useful when omitting the road surface, it has very high risks because it may fail to detect static objects. Another drawback of RADARs compared to LIDARs or cameras is that they have limited vertical resolution, which can cause problems due to the reflections from static objects. Such objects, such as manholes or cans, are referred to as RADAR clutter and are another reason why RADARS usually neglect static objects. As it can be observed from the details given above, the nature of LIDARs and RADARs is complementary, which means that if one sensor fails, the other will step in and accurately detect object instances. If both sensors are functioning, more redundant data for each object will be available, which will mean less uncertainty in the position of each detected instance.

By fusing the complementary information obtained from all the sensors, the scene can be better understood and represented. As it can be observed, each sensor can have at least one point of failure, but the fusion of complementary data can lead to a better, more robust and reliable representation of the environment.

**Figure 1:**
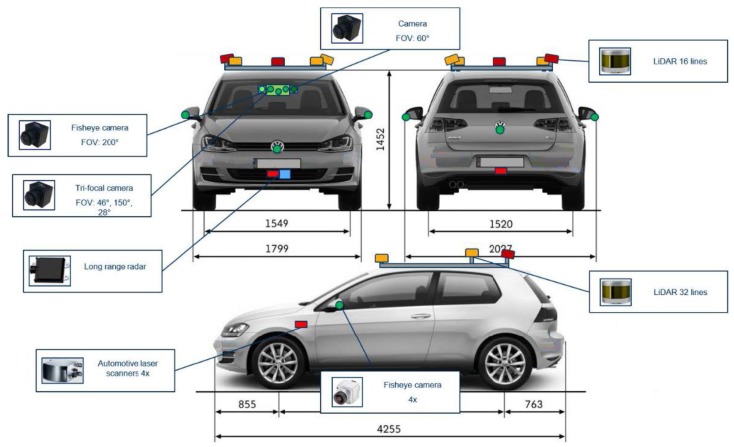
Position of sensors on the ego vehicle.

In the current paper, we fuse the redundant information coming from multiple types of sensors such as: trifocal camera, fisheye camera, 4-layer LIDAR, 16-layer LIDAR and long-range RADAR. The sensor setup and its positioning on the reference vehicle (or ego vehicle) are illustrated in Figure 1. An additional 32-layer LIDAR was mounted on the car but it was not used.

Due to the unpredictable behavior that objects on the road may have in changing weather conditions, the complexity of fusion algorithms has increased [4]. Two main approaches for sensor fusion can be identified in the literature. The first category refers to tightly coupled sensor fusion, which means that information is fused at measurement level and joint features and models are generated at low level. The second category refers to high-level fusion, in which object candidates or tracks are generated for each sensor and they are fused afterwards. For solving the stabilization and validation problem, our solution is the use of a loosely coupled sensor fusion approach.

Our main contributions are:

- We create a novel data association approach for finding the correct correspondences between the trifocal camera objects and fused hypothesis (super-sensor object); the proposed solution uses polar rays to find candidate matching, and based on a decision tree, which takes into consideration object positions and various physical properties, unwanted associations are gradually removed.
- An original data association scheme based on a combination of multiple appearance and motion features is proposed for the tracking process of the 16 L LIDAR sensor objects; appearance features such as object dimension ratio, object area, RGB color histogram, semantic class similarity, orientation and visible façade as well as motion information are aggregated into a cost function, which is used to find the best track–measurement correspondences.
- We propose a fusion architecture by applying a combination of two types of sensor data fusion methods (a model-based approach using the Unscented Kalman Filter and a data-driven approach using a single-layer perceptron), with the purpose of stabilizing the position retrieved from four types of complementary sensors: 4 L LIDAR, 16 L LIDAR, trifocal camera and RADAR. The two types of sensor fusion are used together to better capture the motion of the road objects. Furthermore, data-driven fusion was also used due to the lack of information regarding the performance and parameters of one of the used sensors.
- Finally, a validation scheme is proposed using the semantic segmentation image. The objects that result from the sensor fusion module are projected onto a semantic segmentation image in order to validate the semantic class of the super-sensor objects using a fuzzy logic approach.

The rest of the paper is organized as follows: in Section 2, we review the literature on multi-sensor fusion, data association and tracking. In Section 3, the proposed solution is presented. In Section 4, we evaluate the results using various experiments and compare the data to the ground truth information obtained from a high-precision GPS. Section 5 concludes the paper.

Due to the high complexity of the problem, only the contributions made in the processing pipeline will be presented in this paper.

## 2. Related Work

In Section 2, we present state-of-the-art solutions in the fields of data association, tracking and sensor fusion that could be successfully applied in the automotive field. Data association and multi-target tracking algorithms are presented in Section 2.1. In Section 2.2, we depict multiple-sensor fusion approaches and we classify the presented methods into white box (Section 2.2.1) and black box sensor fusion (Section 2.2.2) approaches depending on the level of user engineering and intervention in the fusion process.

### 2.1. Multi-Target Tracking and Data Association

In the context of autonomous driving, real-time target tracking in clutter is a challenging task. Autonomous vehicles have to filter and predict various parameters of the detected objects in a short amount of time. With the time requirement in mind, several solutions were developed and presented in the literature such as the strongest neighbor filter (SNF) [5] or the nearest neighbor filter (NNF) [6]. The former selects, from a set of validated measurements, the value with the highest intensity as if it were the one that originated from the desired object. On the other hand, the latter selects the measurement closest to a predicted value.

Another approach used in research is the probabilistic data association (PDA) filter [7], which does not rely on a single measurement to estimate the state and error covariance matrix of the state estimate of an object but uses a set of validated measurements. There are multiple variations of the PDA such as the joint PDA [8], used when dealing with multiple targets, or the integrated PDA, in which the data association probability and the track existence are jointly estimated [9]. A more powerful class of data association algorithms is represented by multi-hypothesis trackers (MHT) [10], which associate all the measurements with tracks after the gating and pruning processes have been used to eliminate candidates with low probabilities. To reduce the computational complexity of MHT, probabilistic multi-hypothesis tracking (PMHT) [11] has been developed. This method assigns to each measurement a probability of belonging to each track. Batch maximum-likelihood-PDA (ML-PDA) is another estimator, which computes a global likelihood ratio for multiple sets of scans, which is another efficient solution for target tracking in heavy clutter [12].

Unlike the previous data association methods mentioned, the probability hypothesis density (PHD) filter [13] is a technique based on the random finite set theory, in which the track initialization is included in the tracking algorithm. The PHD filter mainly has two shortcomings: it cannot associate the same target between frames, which is a drawback if the trajectory of different targets is required, and it cannot provide an accurate estimate for the states because of the closely spaced target interference present due to the appearance of clutter. Different alternatives have been proposed based on the PHD filter such as the Gaussian mixture PHD filter [14], where the posterior probability is defined as a Gaussian mixture, or the sequential Monte Carlo PHD filter [15], in which particles are used to approximate the PHD recursion. Other solutions address the problem of multi-object data association and tracking (MOT) by defining dissimilarity measures based on multiple features for identifying the correct associations, then finding the optimal assignments and finally filtering the results. For example, in [16] the authors create a metric for 2D MOT based on motion, appearance, structure and size. Even though it is competitive, this solution only takes into consideration features available in the 2D space and does not consider semantic information. The final dissimilarity cost function is computed for the 2D case, without taking into account the extension of the association metric to the 3D case, where features may violently fluctuate due to synchronization issues or poor object detection. Another method that uses only video information for target tracking is described in [17]. The authors try to reduce the search space for data associations by using a modified hidden Markov model with different spatial constraints. Furthermore, the optimal set of trajectories is identified by applying a dynamic programming approach on a cost edge graph, whose cost is defined through deep features.

A very common algorithm used for producing smoother and more accurate estimates in target tracking is the Kalman Filter (KF) [18,19]. This algorithm has the Bayes filter at its core, and it assumes that the dynamic function and posterior density have a Gaussian distribution and that the measurement and process functions are linear. Taking into account the mentioned assumptions, the filter recursively computes its optimal parameter estimates using its posterior density. The assumptions regarding the linearity of the motion and measurement models are often violated, since in the real world the object motion dynamics and measurement functions can be very complex. Two approaches were introduced in order to cope with this problem: the Extended Kalman Filter (EKF) [20] and the Unscented Kalman Filter (UKF) [21]. The first one employs a linearization based on a first-order Taylor series expansion; however, this type of linearization using Jacobians can be computationally expensive. The second one manages to better approximate the nonlinear function by using the so-called sigma point sampling technique. Depending on the different abstraction levels at which we represent the environment, we can use specific filters.

A method of environment representation that is able to provide lower processing time and higher flexibility is grid mapping. In this type of representation, the world is discretized in cells, and each one contains properties such as occupancy, among others. Various techniques have been proposed to model and track occupancy grids. For example, in [22] a particle filter is used to estimate the grid cell occupancy and speed. The particles are considered independently and have their own position and speed. In [23], a mix of static and dynamic particle filters is employed to estimate object properties.

Due to the high popularity of deep learning, tracking solutions using different deep neural network models have been developed. In [24], Wojke et al. propose an appearance descriptor based on a deep learning approach, which computes the final similarity score using a weighted sum of the Mahalanobis distance and the cosine distance of the appearance vector of the tracklet and measurement. Another tracking approach using deep learning is presented in [25], where the appearance of an object is learned in an offline manner. The deep learning model is trained on thousands of video sequences and does not need to perform any learning at runtime, making it a fast deep-learning solution. The main drawback of deep learning-based algorithms is that they require huge amounts of annotated data. Furthermore, similar objects to those in the training set may get tracked, even though they were not present in the training set. Also, if the training set is unbalanced then some categories may be favored over others. Lastly, since motion information is not included when training the model presented in [25], if an object that is tracked is moving in one direction and it gets partially occluded by a similar object moving in the opposing direction, there is a chance that the tracker will latch onto the wrong object. In [26], the authors propose a neural network model that can localize the exact position of people in a scene. With this information, people can be detected and tracked in dense environments. For achieving a high-performance solution, which outperforms other methods, the authors propose a scale aware network model (DISAM) that takes into account the scale variations of head images, and they perform a non-maximal suppression to get the accurate head positions. Deep learning-based tracking solutions are not applicable in our case due to the nature of our input (we are not receiving the whole image), time requirements, lack of annotated data that can cover all traffic scenarios for multiple classes of objects (such as cars, pedestrians, cyclists, poles, trucks etc.) and high running time when transmitting large amounts of data between processing modules.

### 2.2. Sensor Fusion

Multi-sensor fusion can be achieved at four processing levels, depending on the stage at which the data fusion takes place. The four stages are: signal, pixel, feature and decision-level fusion [19,27]. Depending on the level of visibility and control inside the sensor fusion module, a further taxonomy can be made by splitting the state-of-the-art into white and black box sensor fusion solutions.

#### 2.2.1. White Box Sensor Fusion

Existing algorithms that have had a major impact in the development of the sensor fusion field and revolutionized the automotive industry include solutions for advance tracking [28], optimal filtering [29] and multi-sensor fusion [30]. Current methods in the state-of-the-art [19,31] first detect object instances in the sensor space, and then track and fuse detections using the Kalman filter [19] or a Dempster–Shafer [32] algorithm combination. Other solutions adopt a LIDAR-based approach for generating the object hypothesis and a camera sensor for verifying it [27,33,34]. These methods use geometric features to find 3D region candidates in the LIDAR space [35]. The image patch corresponding to the 3D region and described by the Histogram of Oriented Gradients [36] is then checked by several classifiers. Multi-sensorial methods using LIDARs and cameras outperform any algorithms that use single sensors; however, they do not offer acceptable results in adverse weather conditions. In the work of Chen et al. [37], object detection proposals are generated in the top-down LIDAR view and are projected in front of the LIDAR. All features are fused, and oriented cuboids are extracted. The mentioned solution uses only a single LIDAR setup and assumes that all objects can be localized from the top-down view of the point cloud and that they are situated on the same spatial plane.

#### 2.2.2. Black Box Sensor Fusion

Artificial Neural Networks (ANN) can offer another way of performing sensor data fusion [38]. The image or data features that will be fused are first extracted and normalized. The ANN, through its architecture, is able to approximate any nonlinear function defined by a representative set of training data. After the network has been trained, it can be used to infer information about data that have not been presented to it during the training phase. Thomas et al. [39] created an efficient method for pixel-level fusion, using a fully connected ANN, of low light television cameras (LLTV) and far infrared images (FLIR) with the goal of retaining information of interest from both sensors. Some of the normalized features, which are visible in both FLIR and LLTV cameras, and fed to the network are straight edges, winding edges, anisotropy and contrast information from each image. In [40], the authors propose a novel deep learning-based LIDAR and image fusion neural network (PMNet) for extracting meaningful information from aerial images and 3D point clouds. The fusion procedure uses spatial correspondence—point-wise fusion—which is done at feature level and shows improved performance with low memory usage and less computational parameters. Another example where 2D images are fused with 3D point clouds is illustrated in [41]. The authors propose a network model for an accurate 3D object detection solution by exploiting multiple related tasks such as 2D and 3D object detection, depth completion and ground estimation. The fusion is done, at first, at point and feature levels, and then it is refined with results from the ground plane estimation and depth completion modules. Caltagirone et al. propose a novel fusion deep neural network to integrate LIDAR point clouds and camera images with the purpose of detecting the road surface [42]. The cross-fusion FCN (fully convolutional neural networks) performed better than single-modality methods and other fusion approaches designed for road surface detection, which were presented by the authors. There are numerous artificial neural network solutions and models developed for the task of sensor fusion such as BPNN [43] (back propagation neural net), which has a fusion strategy that uses a fully connected neural network trained with backpropagation and Bayesian inference, SOFM [44] (self-organizing feature maps), a fusion method that uses self-organizing feature maps to fuse data for wireless sensor networks and to form a hierarchical network structure and complete cluster head selection by competitive learning among nodes, or ARTMAP [45] (adaptive resonance theory map), which describes a fusion method with a neural network model that produces one-to-many and many-to-one mappings from input vectors to output classes with the purpose of terrain and object classification from complex and conflicting data.

This paper builds upon the state-of-the-art by proposing a multi-object fusion and validation approach useful in detecting and handling inconsistencies between different sensor measurements, which result from the perception system. Regarding data association and tracking methods, we propose a novel data association approach of associating and tracking 3D objects detected by a 16 L LIDAR sensor. The novel contribution consists of a two-step association algorithm, the extraction and weighted combination of motion and appearance features that may fluctuate due to poor object detection methods, object motion or bad weather conditions. The appearance cost takes into consideration physical properties such as object dimension ratio, object area, RGB color histogram, semantic class similarity, orientation and visible façade information as well as semantic class information. Multiple motion models were used to better describe and capture the motion of objects from the scene. This contribution is detailed in Section 3.4. In Section 3.3, we present another original data association algorithm, which is based on a decision tree and uses polar rays and object characteristics such as object area, visible façade, dimension ratio, and relative position between candidates in different coordinate systems (Cartesian and Polar) to find correspondences between any target objects (such as super-sensor objects or LIDAR objects) and trifocal camera objects. Designing fusion methods based only on deep learning techniques is not practical because, regardless of how many data one uses for training a neural net, there will still be real-world situations that will not be covered by the dataset. Furthermore, due to the fact that some vendors do not offer performance information for their products, and do not allow evaluation of their algorithms, it is difficult to construct the covariance matrices necessary for fusing objects using model-based approaches. For these reasons, the current paper introduces a model and neural net-based approach to fuse data. The fusion architecture that is created combines two types of sensor data fusion methods, using the Unscented Kalman Filter and a single-layer perceptron for reliable merging of information retrieved from five complementary sensors. The details of our method are presented in Section 3.5 and Section 3.6. Finally, in Section 3.7 an original validation method is proposed, which projects the fused objects onto a semantic segmentation image and compares the dominant semantic class from the projection with the semantic class of the super-sensor object.

The input for our solution is offered as cuboids with different properties. Some of them are common for all sensors (width, length, height etc.) while others are sensor-specific (object class coming from the trifocal camera, for example). Given the nature of our input, we will perform high-level fusion using variations of the Kalman algorithm for tracking, filtering and sensor fusion.

## 3. Proposed Solution

### 3.1. General System Overview

**Figure 2:**
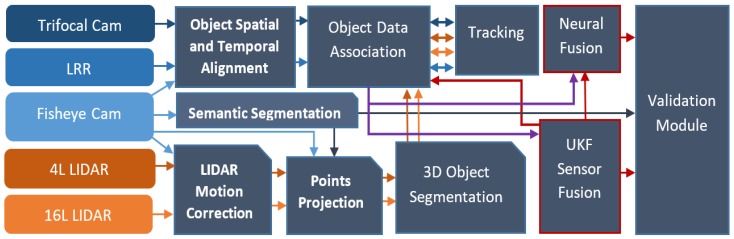
Processing pipeline used in the self-driving car solution. The flow of data from each sensor to the modules is depicted using the sensor or module color assigned.

The main modules of the processing pipeline are presented in Figure 2. Each sensor is colored with a different color, and to symbolize the data flow from specific sensors more intuitively, the arrows that link different modules are depicted with the same color as the sensor. The five complementary sensors used are: the trifocal camera (Trifocal Cam), the long-range RADAR (LRR), the fisheye camera (Fisheye cam), and the 4- and 16-layer LIDARs. Even though a module can take input data from multiple sensors, the algorithms applied on each sensor type may be different; however, for brevity we have given the modules a generic name. In the presented pipeline, the modules Object Spatial and Temporal Alignment and LIDAR Motion Correction deal with the spatio-temporal alignment of raw sensor data to a reference timestamp, given by the front fisheye camera. This alignment is performed at object level for the long-range RADAR and trifocal camera objects, as described in Section 3.2., and at point cloud level in the LIDAR motion correction module [46].

The motion-corrected point clouds are projected, using the Points Projection module, onto the intensity image and onto a semantic segmentation image obtained as described in [47] and given by the Semantic Segmentation module, to obtain an enhanced point cloud where each 3D point will contain semantic information as well as color information. The enhanced point clouds are fed to the 3D Object Segmentation module, where cuboids representing real-world objects are extracted for each of the two LIDAR sensors. The acquired objects from all the sensors are fed into the Object Data Association and Tracking modules where, depending on the sensor input, specific algorithms are applied. In this paper, the original contributions regarding the data association functions for the LIDAR and trifocal camera sensors are discussed in Section 3.3 and Section 3.4. In Section 3.4, the proposed tracking algorithm for the LIDAR objects is also presented. The next function in the pipeline is the sensor fusion of complementary sensorial data. The proposed sensor fusion approach is split into two modules. The UKF Sensor Fusion module is responsible for fusing data coming from the LRR and LIDAR sensors, and it maintains the semantic class coming from the trifocal camera as a parameter (without actually fusing the position of the trifocal objects to the super-sensor objects). The Neural Fusion component merges the trifocal object and the hypothesis generated by the UKF Sensor Fusion module. Its result will be a set of fused object hypotheses. This sensor fusion function is performed in two steps. In the first step, the tracked objects coming from different sensors are associated in the Object Data Association module, and the correspondences are stored in a lookup table for fast access. In the second step, the association of data coming from complementary sensors is performed with the generated fused UKF object hypothesis from the first step. Tracked objects that are not associated with any hypothesis in the second step are associated according to the first step. The sensor fusion modules have the purpose of stabilizing object parameters such as speed and position, among others. The neural fusion has the purpose of generating a new validation hypothesis for every UKF-fused object. The output of the association between objects from different sensors that is achieved in the first step is depicted with a purple color arrow in Figure 2. The bidirectional arrows between the Data Association and Tracking components refer to the fact that raw data are first passed to the tracking module and the resulting tracked objects will be used in the sensor fusion step. The tracked objects are associated and merged according to the two steps of the fusion module. The main functions from the fusion modules are detailed in Section 3.5 and Section 3.6.

Following the sensor fusion component, we propose a novel Validation Module, which has the purpose of validating the class and position of the generated 3D cuboids using the semantic segmentation image, generated by the Semantic Segmentation module. The validation module is presented in Section 3.7. The proposed solution has been implemented keeping in mind the low running time requirement and need for low resource consumption.

### 3.2. Object Spatial and Temporal Alignment

The first step in our processing pipeline consists of the spatial and temporal alignment of the data. Temporal alignment is achieved by selecting the sensor frame that has a timestamp that is the closest (has the smallest difference) to a reference timestamp. For computing the motion correction, we must first take into account what is happening in the reference frame of the target vehicle. Since we only get the relative speed of a vehicle from a sensor ($V_{relx},V_{rely})$, we have to compute its absolute speed ($V_{x},\;V_{y}$) in both longitudinal (*x*) and lateral (*y*) directions (1). The ego speed on the *x* and *y* axes is denoted by $V_{0x},V_{0y}$.

(1) $$V_{x}=V_{relx}+V_{0x}\;V_{y}=V_{rely}+V_{0y}$$

After computing the absolute object speed, we have to consider the position (*x*, *y*) of the target vehicle after its movement in a time interval Δt in the ego vehicle reference frame. This can be simply achieved by using the motion laws depicted in (2), where ($xt_{0},\;yt_{0}$) represents the initial position of the target vehicle before the movement:

(2) $$V_{y}=V_{rely}+V_{0y}\;y=yt_{0}+V_{y}\Delta t$$

We will now take into account what is happening with the ego vehicle during the movement. We consider that the ego vehicle is moving from point *A* to point *B* on a distance *S*. The ego vehicle sweeps an angle θ, taking into account the different positions in which it moves in a time interval Δt. The θ angle can be expressed using the yaw rate ϕ of the ego vehicle (3).

(3) θ=ϕΔt

The resulting speed $V_{0}$ of the ego vehicle is composed of the speed components in the *x* and *y* directions ($v_{0x},\;v_{0y})$ (4).

(4) $$V_{0}=\;\sqrt{v_{0x}^{2}+v_{0y}^{2}}$$

Finally, the displacement *S* is computed as illustrated in (5).

(5) $$S=V_{0}\;\Delta t$$

If the θ angle is smaller than a predefined small threshold, we consider that the motion performed is in a straight line, otherwise we consider the vehicle to be moving on a circle chord, with radius *R* and sector angle θ.

The distance *S* can be computed in two ways. The first method is depicted in Equation (5) and the second in (6).

(6) S=ΘR

**Figure 3:**
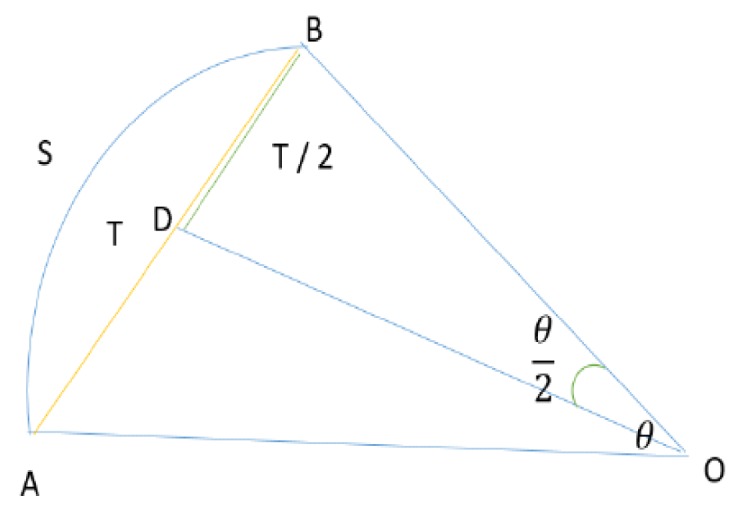
Notations (on the figure) depicting ego vehicle movement from point A to point B.

Taking into account Figure 3, we observe that in the right triangle ODB we can determine the expression of *T*/2, where the radius *R* is equal to the length of segments OB and OA.

The final expression of *T* can be identified by combining Equations (5)–(7).

(7) $$\frac{T}{2}=R\sin\left(\frac{\Theta }{2}\right)$$

(8) $$T=\frac{2\left(V_{0}\Delta t\right)}{\Theta }\sin\left(\frac{\Theta }{2}\right)$$

**Figure 4:**
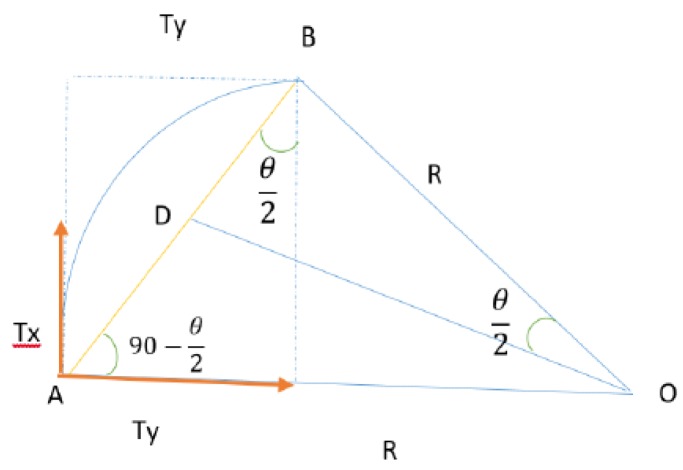
Tx and Ty movement components.

If the ego vehicle has a straight (rectilinear) movement, the classical motion equation can be used, otherwise Equation (8) can be applied. Having computed the distance *T* on which the ego vehicle has moved in time ∆*t*, we can compute the *x* and *y* components of the movement (*Tx*, *Ty*), as illustrated in Figure 4 and expressed analytically in Equation (9).

(9) $$Ty=T\sin\left(\frac{\Theta }{2}\right)\;Tx=T\cos\left(\frac{\Theta }{2}\right)$$

The last step of the object motion correction is finding the position of the target vehicle in the current ego vehicle reference frame (point B). The final positions using rotation and translations are depicted in Equations (10) and (11).

(10) $$\left[\begin{array}{c} x\\ y \end{array}\right]=\;\left[\begin{array}{cc} \cos\left(\Theta \right) & -\sin\left(\Theta \right)\\ \sin\left(\Theta \right) & \cos\left(\Theta \right) \end{array}\right]\left[\begin{array}{c} y-Ty\\ x-Tx \end{array}\right]$$

(11) $$\left[\begin{array}{c} x\\ y \end{array}\right]=\;\left[\begin{array}{c} \left(y-Ty\right)\cos\left(\Theta \right)-\left(x-Tx\right)\sin\left(\Theta \right)\\ \left(y-Ty\right)\sin\left(\Theta \right)+\left(x-Tx\right)\cos\left(\Theta \right) \end{array}\right]$$

### 3.3. Data Association for the Trifocal Sensor

Data association is an important step in the sensor fusion pipeline. In this stage, we correlate similar data that come from multiple sensors with the purpose of enriching the available information for the detected objects. Associating measurements coming from the trifocal camera to super-sensor objects (or hypotheses) is a difficult endeavor chiefly due to the fact that the trifocal camera offers extremely poor estimates regarding the position of an object. For the trifocal sensor used, rules or parameters describing the error covariance matrix are not available.

**Figure 5:**
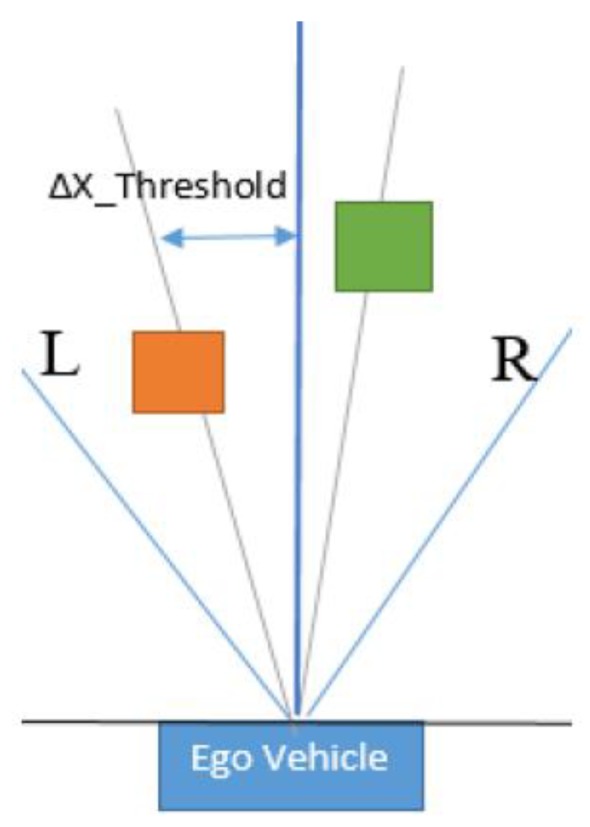
Corresponding objects in the left half (orange) and the right half (green) spaces.

To this end, an association scheme that has the structure of a decision tree has been implemented in order to efficiently associate measurements from the trifocal sensor with target objects. We have tried to exploit as many features for each object as we could, without burdening the real-time performance of the solution. To simplify the problem, virtual 2D objects were created from the motion-corrected 3D bodies, and they were projected onto a virtual 2D image space. From the entire space, we only selected objects that are at most 50 m in front of the vehicle and 20 m to the left and right sides. The search space is divided into two sides: the left and right hand sides. Each object is assigned to a side, and when the algorithm searches for an object on one hand side, it also takes into consideration that its correspondence could be situated at the border of the other hand side. For example, for an object situated on the right-hand side, a search is performed on the left-hand side up to a certain distance threshold on the *x* axis. This is exemplified in Figure 5 for better understanding. After finding measurements that are in proximity to the target object, the cuboids are filtered further by taking into consideration the object dimensions. For each hypothesis, a search among the filtered measurements is performed to identify the one that has the most similar visible façade to that of the target object. This issue is solved by computing the difference between the target object’s visible façade and the potential trifocal object’s visible façade. In this manner, the best correspondence between hypothesis and measurement with respect to the most similar, visible façade is found. For further processing, we also keep objects at a dimension difference within a certain tolerance limit with respect to the best-found difference, since objects may have different properties due to sensor measurement errors. The tolerance parameter, denoted by *ζ*, was determined experimentally and set to 5 pixels. The candidate objects whose difference is not in the interval dictated by the identified minimal difference are eliminated according to Equation (12).

The parameter ∂ in Equation (12) denotes the dimension difference, and *δ* denotes the minimal found difference.

(12) $$f\left(\partial \right)=\;\{\begin{array}{c} 1,\;if\;\partial \;\in \left[\delta -\zeta ,\delta +\zeta \right]\;\\ \;0,\;otherwise\; \end{array}$$

Another physical property exploited in our architecture is the object area. In our solution, a trifocal object is deemed suitable for further investigation if the ratio between the target object and the trifocal object areas is lower than a pre-defined threshold *η*. This part has the purpose of removing hypotheses that are much smaller than the target object. Moreover, similar objects are identified by sweeping through the whole list of objects using a polar ray with a fixed position in the central region of the ego vehicle. The conversion from Cartesian coordinates to polar coordinates is done using the expressions from (13) below.

(13) $$\rho =\sqrt{x^{2}+y^{2}}\;\Theta =\tan^{-1}\left(\frac{y}{x}\right)\;$$

**Figure 6:**
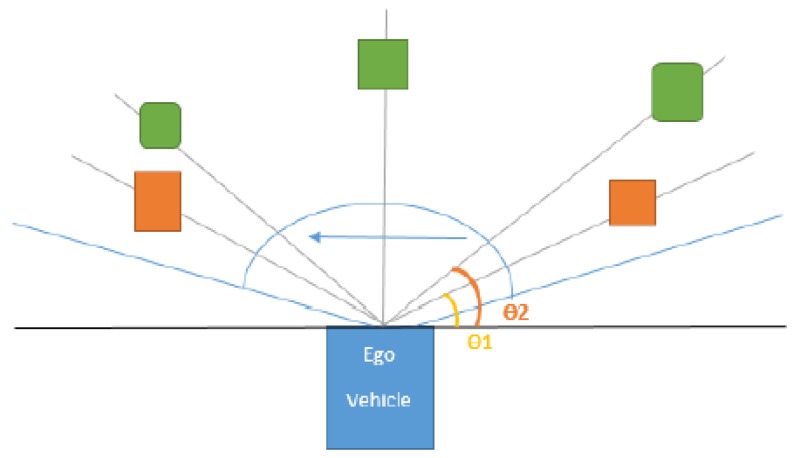
2D sweeping polar rays for object association are depicted in a light blue color. In orange, we illustrate motion-corrected trifocal objects, and in green, the target objects.

The process of finding associations is depicted in Figure 4, where the green squares represent target objects, the orange squares represent trifocal measurements, and the blue square in the middle is the position of the ego vehicle. We have represented the polar rays with a gray color. Using this methodology, we observe that corresponding objects are situated in proximity to one another, with the smallest difference between the corresponding Θ angles and the smallest distances ($\rho_{1}-\rho_{2}$ and $\Theta_{1}-\Theta_{2}$ are small for similar objects). Therefore, we further filter the potential trifocal objects by considering just the items that have a difference in the range of ρ and Θ angles, with respect to the target object, smaller than a set of predefined thresholds *(*$\Theta_{t}$ and $\rho_{t}$). An intuitive depiction of this mechanism is presented in Figure 6.

Finally, for the remaining filtered objects, we associate the target object to the trifocal measurement closest to it, taking as a distance metric the Euclidian length. The system variables are updated each time a potential correspondence is found. The algorithm offers as output the best-found association for hypothesis and trifocal objects. In the image below, we have included the results of the association on a single-vehicle detection. The algorithm also works for multiple objects; however, for clearness and better understanding, we have demonstrated the working of our solution on a single image. The algorithm can successfully associate trifocal measurements with any kind of target objects (super-sensor objects, LIDAR objects or RADAR objects).

**Figure 7:**
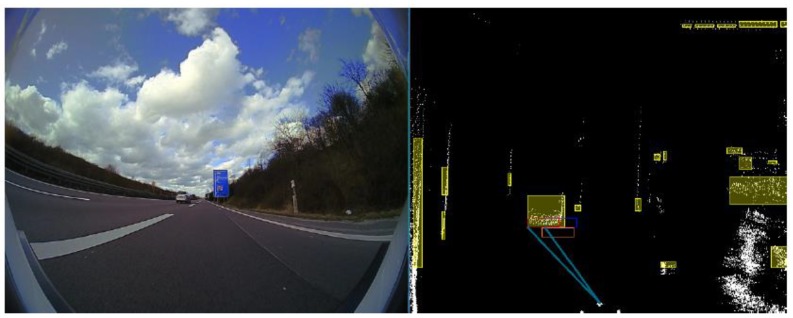
Results of LIDAR object to trifocal object association. On the left-hand side, the color image of the recorded scene is displayed. On the right-hand side, the processed sensory data containing trifocal and LIDAR objects are shown.

In Figure 7, on the right-hand side, in white we represent the fused 3D points coming from 4 L and 16 L LIDARs, the yellow squares represent filtered LIDAR objects, in orange we represent the original trifocal object position, in dark blue the motion-corrected trifocal position value, and in red the associated position for the trifocal object. The intensity image on the left-hand side represents the real-world scene. The polar association rays are depicted in a light blue color in this frame. In the situation in which there is no suitable correspondence for an object, the association will return the motion-corrected position for the trifocal object.

**Figure 8:**
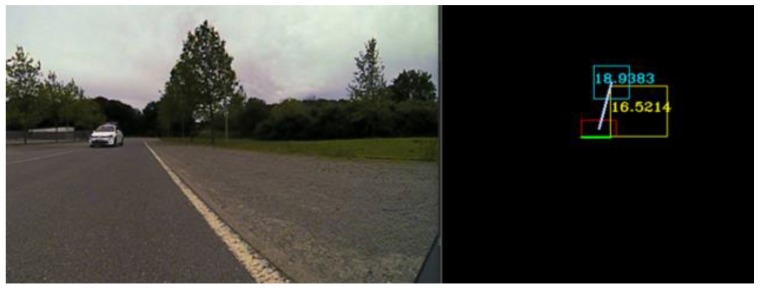
Correspondence of the trifocal camera objects and LIDAR and RADAR sensor measurements. On the left-hand side, the color image captured by the camera is shown. On the right-hand image, the data association of objects from different sensors is illustrated.

In Figure 8, we have used the same algorithm to find the correspondences between the LIDAR, RADAR and trifocal objects. The LIDAR object is represented in yellow, the RADAR object is colored in cyan, and the trifocal object is depicted in red. On the left-hand side, the real-world environment observed by the sensors is illustrated. The constants used in the trifocal data association were determined experimentally, and their values are: *X_Threshold* = 25$,\;\zeta =5,\;\eta =1,\;\Theta_{t}=5,\;\;\rho_{t}=5$.

### 3.4. Data Association and Tracking of the 16 L LIDAR Objects

The 16 L LIDAR objects are obtained from the 3D point cloud using a custom segmentation algorithm, which is not discussed in this paper. Before performing the sensor fusion task, the 3D objects, which come from an earlier module in the pipeline, should have a filtered position, velocity and unique ID assigned to each of them. A two-step tracking algorithm based on [48] is used. The 3D objects are transformed into 2D virtual objects, embedding all properties of the original 3D cuboids: the original *x*, *y*, *z* position, the width and length of the original object, the *x*, *y* positions in the 2D grid, the width and length of the object in the 2D grid, the top three semantic classes, the marginal probabilities of each of the classes, and an eight-bin color histogram for each of the three channels (R, G, B). The most probable three semantic classes and their frequencies were included mainly due to the fact that during the point cloud segmentation process and cuboid formation, each 3D point had a semantic class associated with it. The semantic image, even though it is of high quality, can often have errors and include undesired classes.

**Figure 9:**
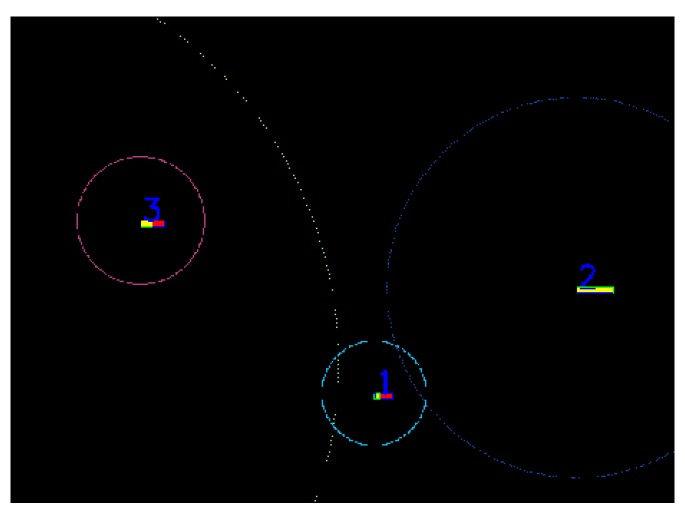
First step of the data association algorithm. In this image, three detected objects and their tracks along with their covariance ellipses are shown.

In the first step, the 3D virtual objects are projected onto a 2D color grid. In this grid, the cells occupied by the LIDAR objects (measurements) have the red channel set to 255, and the other channels contain the position of the object in the original virtual object list. The tracked objects (hypotheses) are also converted to virtual objects and projected on the same virtual grid, but colored in white. The intersection area between the two types of objects is depicted in a yellow color. Figure 9 illustrates the intersection between tracks and measurements, as well as the covariance ellipses of the tracks.

**Figure 10:**
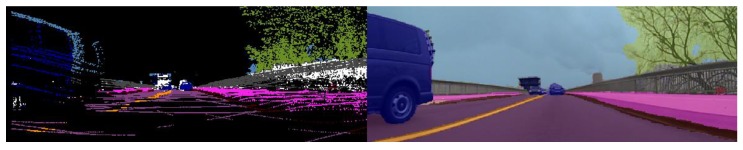
Semantic class of each 3D point. On the left-hand side, we observe the assigned semantic class of each projected 3D LIDAR point. In the right image, the semantic class image with 50% transparency is overlapped over the color image.

It may happen that multiple measurements fall in the covariance ellipse of a hypothesis. To calculate the posterior distribution of a hypothesis, we search for the measurement that is the most similar to the target hypothesis. Exploiting all the available information, we define several similarity scores described analytically by Equations (14)–(17). Each similarity score is not reliable by itself due to errors that are propagated from previous stages of the processing pipeline such as motion correction errors, point projection errors, point cloud segmentation errors and semantic segmentation errors. For this reason, the similarity measure used will encapsulate and take into consideration all the measured scores. In Figure 10, an illustration of the semantic class of each 3D point is shown. It can clearly be observed on the passing vehicle that due to object motion, not all the points corresponding to a 3D cuboid receive an appropriate semantic class.

Geometric properties such as visible façade, object area and measurement–hypothesis overlapping are used as described in Equations (14) and (15) for eliminating candidates that are not similar to the compared object. We denote by $A_{M}$ and $A_{T}$ the areas of the LIDAR measurement and of the tracking hypothesis. We denote by $T_{f}$ and $L_{f}$ the most visible façade of the track and LIDAR object. The overlapping area of the track and measurement is denoted by *η*. The object dimension ratio is symbolized by *ε*. With β, we represent the similarity with respect to area and visible façade.

(14) $$\varepsilon =\frac{\eta }{\left|A_{M}-A_{T}\right|}$$

(15) $$\beta =\left|A_{M}-A_{T}\right|+\left|L_{f}-\;T_{f}\right|$$

Another way of comparing objects is based on color similarity. Each object has embedded into it a reduced three-channel color histogram that has eight bins per channel, and it is obtained by projecting the 3D points that correspond to an object onto the front RGB image. The 3D points that fall inside the image cast a vote in a specific bin from a channel of the object’s color histogram. Each bin of the histogram can store 32 intensity values. The root mean square (RMS) metric is used to compute the color difference between a track and a measurement. Analytically, this difference is presented in Equation (16), where n is the number of bins of the color histogram for each channel. The RMS error is computed for all channels.

(16) $$\Omega =\frac{1}{n}\sum_{j=1}^{3}\sum_{i=0}^{n}{\left(veloHist_{j}\left(i\right)-trackHist_{j}\left(i\right)\right)}^{2}$$

The semantic class of a measurement is obtained by projecting the point cloud that corresponds to a measurement onto a semantically segmented image, computed by using the ERF (Efficient Residual Factorized) neural net presented in [47]. Due to calibration and motion errors, the points that correspond to an object may not fall precisely on the desired object (Figure 10); therefore, the most probable three semantic classes with their corresponding probabilities are extracted according to (17).

(17) $$\varphi =\;\overset{3}{\underset{i=0}{\sum }}\{\begin{array}{c} 0,\;if\;w\left(i\right)=\;-1\;\;\;\;\;\;\;\;\;\;\;\;\;\;\;\;\;\;\;\;\;\;\;\;\;\;\;\;\;\;\;\;\;\;\;\;\;\;\;\;\;\;\;\;\;\;\;\;\;\;\;\;\;\;\;\;\;\;\;\;\;\;\;\;\;\;\;\;\;\;\;\;\;\;\;\\ \left|Measurement.P\left(i\right)-Hypothesis.P\left(w\left(i\right)\right)\right|,\;otherwise \end{array}$$

In Equation (17), the semantic similarity score is denoted by φ. The variable *w*(*i*) takes the value of the position where the semantic class of the hypothesis matches the semantic class of the measurement. If there is no semantic class available in the target that should match the semantic class of the measurement, *w*(*i*) will take the value −1. The absolute value of a variable *a* is denoted as |*a*|. The final association score is obtained as shown in Equation (18).

(18) $$AgSc_{1}=\;\alpha \;wd+\left(1-\;\alpha \right)\left(\epsilon +\beta +\;\Omega +\;\varphi \right)$$

The constant α represents a weighting factor and has been set to 0.2. We denote with the variable *wd* the Euclidean distance between two objects.

After selecting the measurement that minimizes the score computed in (18), the object corresponding to that measurement is marked as used so that it will not be considered for any further association. If the first association step does not offer a result with a good enough association probability, the second step of the association scheme is performed. In the second step, for each unassociated tracked object, a search for a potential measurement is performed in a larger covariance ellipse. The geometric properties and distance of the LIDAR and tracked objects are again verified. Quantities that are not included have a tendency of fluctuating much more than the geometric properties. A LIDAR object gets associated to a track if it minimizes the score from (19). The second step of the data association procedure is useful for making associations with objects that have sporadic behavior and fluctuating features, such as pedestrians.

(19) $$AgSc_{2}=\;\alpha \;wd+\left(1-\;\alpha \right)\left(\epsilon +\beta \right)$$

**Figure 11:**
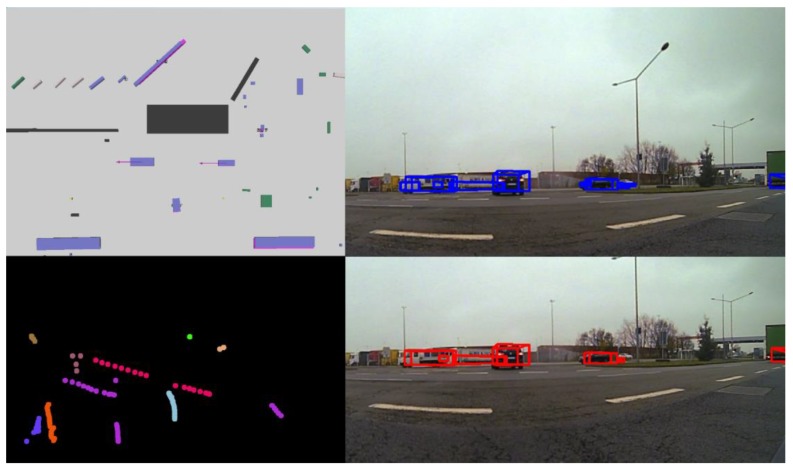
Tracking of dynamic and static objects. In the top-left corner, the birds-eye view 3D image of the scene can be observed. In the top-right corner, the 3D object detections are projected over the color image in blue. In the bottom-right image, the tracks corresponding to the detections are shown in red. In the bottom-left image, the traces of the tracked objects are shown in the same color for the same object instance.

**Figure 12:**
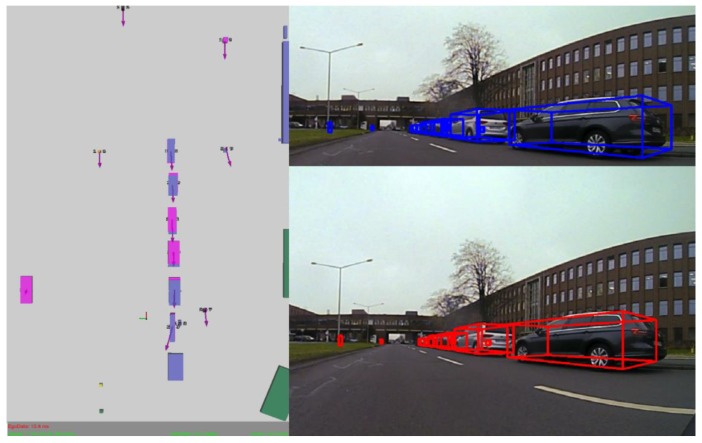
Tracking of static objects. In the left image, the 3D top-view scene containing measurements and tracks with their relative motion vectors is shown. In the top-right and bottom-right images, the measurements and tracks are projected onto the RGB image in blue and red, respectively.

The results of the tracking algorithm with the previously proposed association metric are illustrated in Figure 11. The cuboids corresponding to the tracked objects in the scene are displayed in red color in the bottom-right image. In the top-right part of Figure 11, we depict the cuboids corresponding to the measurements in blue. In the top-left part, all the detected objects are illustrated in a top-view image. The color of each cuboid denotes the semantic class of that object. The pink cuboids that are beneath some objects represent the tracked objects. In the current tracking algorithm, we track objects with the following semantic classes: person, rider, car, truck, bus, train, motorcycle, bicycle, pole and traffic sign. We can observe the motion vector for the two cars that are passing in front of the ego vehicle. The ego vehicle is waiting in the intersection; since the car is not moving, static objects do not have a visible motion vector. In the bottom-left part of Figure 11, the object ID and history are depicted using different colors for each instance. In Figure 12, we illustrate the results of tracking static vehicles. On the left-hand side, we observe the 3D tracked objects in pink, each having an associated motion vector and a unique ID, and in other colors, other measurements are represented. In this image, the position of the ego vehicle is situated where the small green and red cross is located in the middle.

The reason for sometimes seeing the tracked object over the measurement and other times the measurement over the tracked object is due to the ADTF viewer [50]. On the right-hand side, similar to Figure 11, we see the tracks in red color and the measurements in blue color projected onto the front image.

**Figure 13:**
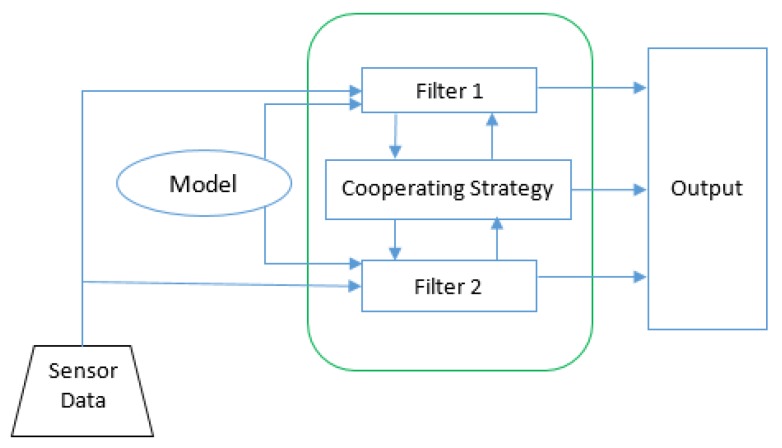
Graphical depiction of the tracking module with two motion models.

The proposed two-step data association and tracking procedure based on the two motion model filters, which can be seen in Figure 13, is an improvement of the algorithm presented in [48]. In this paper, the authors implemented a two-step tracking algorithm, which uses a decision tree to find the best associations between tracks and measurements. Candidate measurements are gradually removed based on the differences in features such as object dimensions, overlapping area and distance between the track and measurement, until the most probable item is left. Furthermore, two motion models, the constant velocity and the constant turn rate and velocity models, are used and combined to solve the motion uncertainty issue. One of the main downsides to the association method used in [48] is that it does not exploit all appearance information that could be gathered for an instance, and motion information is not exploited at all. Furthermore, to find the best global solution, in the current paper we have used a cost-based approach and extracted the best associations by using an optimization algorithm called the Munkres assignment algorithm.

### 3.5. The Unscented Kalman Filter and Sensor Fusion

In the sensor fusion module, we accumulate the information recorded by multiple sensors in one object as if the data were provided by a super-sensor. Considering that the sensor, measurement and motion models are linear and Gaussian, the exact posterior density can be expressed as a Gaussian mixture with one term for every association at time *k*, as seen in Equation (20). The term $w_{k|k}^{\theta_{1:k}}$ is a probability mass function that denotes the probability of association to a measurement, and $P_{k|k}^{\theta_{1:k}}$ represents a probability density function. We denote the fact that the Gaussian mixture spans over all associations that fall in the covariance ellipse of a target by the sum $\underset{\theta_{1:k}\;}{\sum }.$

(20) $$P_{k|k}\left(X_{k}\right)=\;\overset{nrOfSensors}{\underset{i=0}{\sum }}\underset{\theta_{1:k}\;}{\sum }w_{k|k}^{\theta_{1:k}}P_{k|k}^{\theta_{1:k}}\left(X_{k}\right)$$

In each update, we try to find the best measurement association for a target $\theta^{*}$ coming from each sensor and prune all other associations that are situated in the covariance ellipse of the target fused object. Finding a single association from each different sensor will give a computationally cheap algorithm that can meet the real-time performance requirement of a self-driving car. The posterior density can be approximated by $P_{K|K}^{Fused}\left(X_{k}\right)$ in (21), where $\theta_{1:k}^{*}$ is the sequence of optimal data associations from time 1 to time *k* coming from each sensor.

(21) $$P_{K|K}^{Fused}\left(X_{k}\right)=\overset{nrOfSensors}{\underset{i=0}{\sum }}P_{K|K}^{\theta_{1:k}^{*}}\left(X_{k}\right)$$

For performing the prediction and update steps, we use the CTRV(Controlled Turn Rate and Velocity) motion model and the Unscented Kalman Filter. After the data association step, the sensor measurements that correspond to the same objects are kept in a lookup table. They are successively fed into the UKF in order to obtain the filtered position and velocity information and accumulate all required sensor information in one place. The state vector of our model can be seen in the following Equation (22).

(22) $$\;X_{k}={\left[\begin{array}{ccc} px & py & \begin{array}{cc} v & \begin{array}{cc} \Psi & \dot{\Psi } \end{array} \end{array} \end{array}\right]}^{T}$$

The CTRV motion model has two analytical expressions depending on whether the vehicle is taking a turn or moving in a straight line. An illustration of the CTRV process model when the vehicle is turning is depicted in Equation (23) as follows:

(23) $$X_{k+1}=X_{k}+\left[\begin{array}{c} \begin{array}{c} \begin{array}{c} \frac{v_{k}}{\dot{\Psi_{k}}}\left(\sin\left(\Psi_{k}+\;\dot{\Psi_{k}}\;\Delta t\right)-\sin\left(\Psi_{k}\right)\right)\\ \frac{v_{k}}{\dot{\Psi_{k}}}\left(-\cos\left(\Psi_{k}+\;\dot{\Psi_{k}}\;\Delta t\right)+\sin\left(\Psi_{k}\right)\right) \end{array}\\ 0 \end{array}\\ \dot{\Psi_{k}}\;\Delta t\\ 0 \end{array}\right]+\;\left[\begin{array}{c} \begin{array}{c} \begin{array}{c} \frac{1}{2}{\left(\Delta t\right)}^{2}\cos\left(\Psi_{k}\right)\gamma_{a,k}\\ \frac{1}{2}{\left(\Delta t\right)}^{2}\sin\left(\Psi_{k}\right)\gamma_{a,k} \end{array}\\ \Delta t\gamma_{a,k} \end{array}\\ \frac{1}{2}{\left(\Delta t\right)}^{2}\gamma_{\ddot{\Psi },k}\\ \Delta t\gamma_{\ddot{\Psi },k} \end{array}\right]$$

When the vehicle has a rectilinear motion, the process model has the analytical expression illustrated in (24).

(24) $$X_{k+1}=X_{k}+\;\left[\begin{array}{c} \begin{array}{c} \begin{array}{c} v_{k}\cos\left(\Psi_{k}\right)\Delta t\\ v_{k}\sin\left(\Psi_{k}\right)\Delta t \end{array}\\ 0 \end{array}\\ \dot{\Psi }\Delta t\\ 0 \end{array}\right]+\left[\begin{array}{c} \begin{array}{c} \begin{array}{c} \frac{1}{2}{\left(\Delta t\right)}^{2}\cos\left(\Psi_{k}\right)\gamma_{a,k}\\ \frac{1}{2}{\left(\Delta t\right)}^{2}\sin\left(\Psi_{k}\right)\gamma_{a,k} \end{array}\\ \Delta t\gamma_{a,k} \end{array}\\ \frac{1}{2}{\left(\Delta t\right)}^{2}\gamma_{\ddot{\Psi },k}\\ \Delta t\gamma_{\ddot{\Psi },k} \end{array}\right]$$

The UKF generates a set of sigma points and then propagates them through the non-linear process function. The Gaussian can then be recovered from the newly transformed points. The first sigma point is the mean (25).

(25) $$X_{K|K}^{0}=\;X_{K|K}^{*}$$

The rest of the points are generated with a spreading factor of λ around the mean, as depicted in (26) and (27).

(26) $$X_{K|K}^{i}=\;X_{K|K}^{*}+\;\sqrt{\left(\lambda +n_{x}\right)P_{\left(k|k\right)}\;}$$

(27) $$X_{K|K}^{i}=\;X_{K|K}^{*}-\;\sqrt{\left(\lambda +n_{x}\right)P_{\left(k|k\right)}\;}$$

The resulting probability density function is an approximation of the Gaussian distribution. Even though the UKF is not an optimal algorithm, it is used widely in LIDAR MOT due to its low computational complexity in comparison to the KF.

The covariance matrix is then recovered using the sigma points. To achieve this, the spreading of the sigma points is inverted by using a set of weights, (28) and (29). As can be seen, the weights depend on the spreading parameter λ.

(28) $$w_{i}=\;\frac{\lambda }{\lambda +n_{a}}\;,\;i=0$$

(29) $$w_{i}=\;\frac{1}{2\left(\lambda +n_{a}\right)}\;,\;i=2,\;\ldots ,\;n_{a}$$

The mean and covariance are predicted using equations (30) and (31) below.

(30) $$X_{k+1|K}=\;\overset{n_{\sigma }}{\underset{i=1}{\sum }}w_{i}X_{k+1|k,\;i}$$

(31) $$P_{k+1|K}=\;\overset{n_{\sigma }}{\underset{i=1}{\sum }}w_{i}(X_{k+1|k,\;i}-x_{k+1|k})\;{\left(X_{k+1|k,\;i}-x_{k+1|k}\right)}^{T}\;$$

In the update step, since the measurement models are linear, no linearization procedure is necessary. The Kalman gain is computed based on Equation (32).

(32) $$K=P_{k}H^{T}{\left(\;HP_{k}H^{T}+R\right)}^{-1}$$

In Equation (33), we update the state and covariance matrix based on each sensor measurement reading.

(33) $$X_{k}=X_{k}+K\left(z_{k}-HX_{k}\right)\;P_{k}=\left(I-KH\right)P_{k}$$

For the first frame, associations among all objects that come from sensors are made. The associated objects are kept in two lookup tables in order to make the fusion procedure more efficient. The associated objects are fused for the first frame. After the first frame, the same data association procedure presented above is used for associating the trifocal data with the fused and filtered objects. The LIDAR and RADAR measurements are associated with the fused objects. We take a weighted combination between candidate object area and Euclidean distance, based on Equations (34)–(36). The candidate position in the *x* and *y* dimensions is denoted by *c_x_* and *c_y_*, and the fused object position is denoted by *f_x_* and *f_y_*. The Euclidean distance between the two objects is denoted by *d*. The fused object area is denoted by $F_{A}$, and the candidate object area is denoted by $C_{A}$.

(34) $$d=\sqrt{{\left(c_{x}-f_{x}\right)}^{2}+{\left(c_{y}-f_{y}\right)}^{2}}$$

(35) $$dims=\frac{F_{A}}{C_{A}}$$

(36) $$rez=\frac{\left(0.6*d+0.4*dims\right)}{dims+d}$$

**Figure 14:**
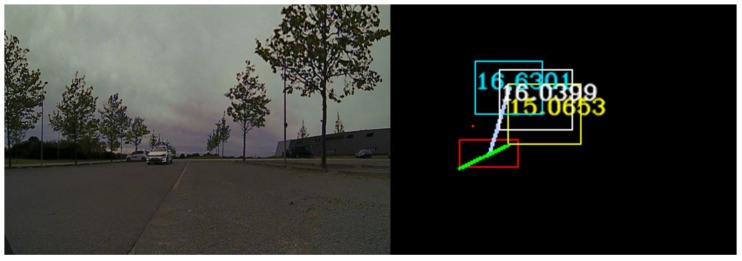
Associations between measurements coming from different sensors and their fusion. On the left-hand side, we observe the RGB image. On the right-hand side, we observe the data associations among sensors as well as the super-sensor object depicted in white.

The same methodology as the one described above is used when performing the data association between the fused object and trifocal data. Each fused object contains a class frequency vector. When a trifocal object is associated with a fused object, it casts a vote at the position corresponding to the semantic class in the frequency vector. Fused objects are kept alive as long as measurements are associated with them. The number of fused objects is kept under control by following a similar procedure as the one we have used in the 16 L LIDAR object tracking. If there are no associations for a number of 10 frames or there is no association for two seconds, the object is destroyed. The fused object is displayed in white in Figure 14, and the other rectangles correspond to measurements coming from other sensors.

### 3.6. Trifocal Camera Object Sensor Fusion

**Figure 15:**
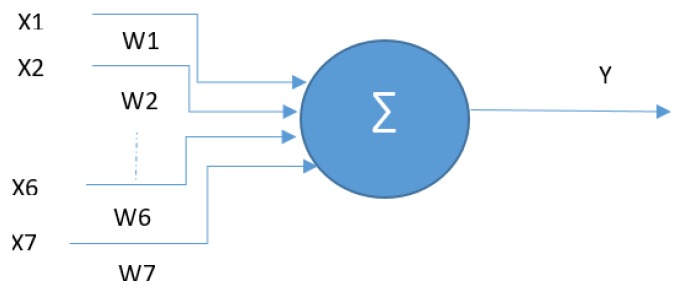
Single-layer perceptron model, with 7 inputs, 1 processing unit and 1 output.

For fusing the trifocal objects, a neural network approach is considered. Such a fusion method has been chosen because there are no available parameters that would allow us to build a measurement covariance matrix for the trifocal objects. For generating ground truth data, a reference vehicle with a very precise GPS, with 2 cm accuracy, is used. Multiple sequences were recorded in various controlled and real-world scenarios. For the fusion task, multiple neural network models were considered; however, a single-layer perceptron model proved to work the best in our scenario. The network has been trained for 500 epochs or until the learning error becomes smaller than 0.1, the learning rate is 0.0001 and no momentum is used. The model has seven inputs and one output, as seen in Figure 15.

The seven inputs are the following: fused object *x* position, fused object *y* position, fused object velocity, trifocal object x position, trifocal object y position, trifocal object velocity *x* and trifocal object velocity *y*. By fused object, we are referring to the LIDAR and RADAR objects fused in the previous step. The transfer function is depicted in Equation (37) below.

(37) $$y=f\left(x\right)=\;\sum w_{i}X_{i}$$

The model was trained using the delta rule (38), with the GPS position of the target vehicle as the reference data.

(38) $$w=\;\Delta w+w_{old}=\;w_{old}+\;\eta \delta x$$

where δ is defined in (39)

(39) $$\delta =y_{target}-y$$

**Figure 16:**
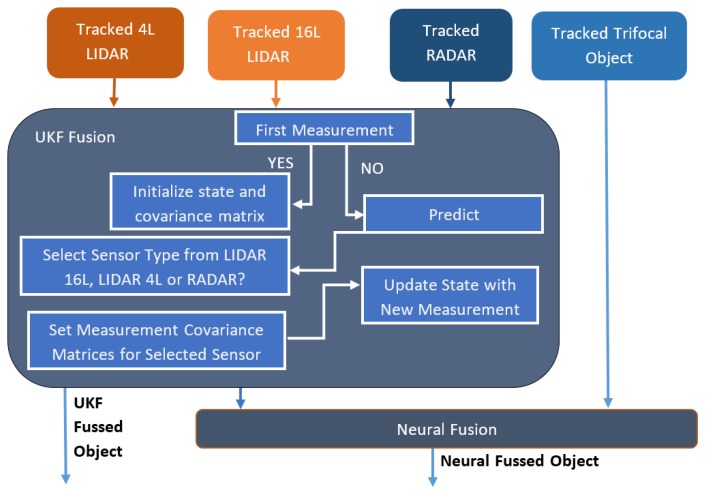
UKF (Unscented Kalman Filter) and neural fusion architecture.

The multimodal sensor fusion architecture is illustrated in Figure 16. The data from each sensor are tracked and filtered before they are introduced into the sensor fusion module.

**Figure 17:**
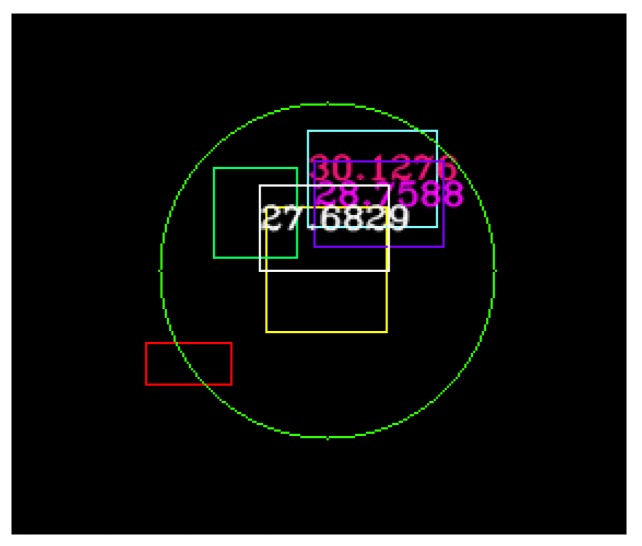
The result of the UKF and neural fusion depicted in white and purple.

The resulting object position obtained after fusion is more stable and can be used with higher confidence for other processing functions. In Figure 17, multiple detections for the same object can be seen. The red rectangle represents the trifocal object, the yellow rectangle represents the LIDAR object, the green rectangle represents the RADAR position, the white object is the fused LIDAR and RADAR, the cyan square with reddish text represents the precise target position obtained from the GPS, and the purple square with pink text is the fused neural model result. As can be seen even from the image, the resulting fused object has a position closer to the target than just the LIDAR and RADAR fused object. The fused object is at a position of 27.68 m, the target is at 30.12 m, while the resulting fused object is at 28.75 m.

### 3.7. Validation Procedure

For validating the fused objects, we use the object’s semantic class that comes from the trifocal objects and the segmented image that results from applying the ERF neural network [47] on the undistorted color image. The first step in the validation procedure is to project the three-dimensional position of the cuboid in the segmented image in order to generate the region of interest. Then, we try to identify the dominant class in the region of interest. For this purpose, we consider a fuzzy approach, where the number of linguistic variables corresponds to the number of object classes. The histogram of the semantic pixel classes is computed within the region of interest, and then the results are normalized using the ROI (Region Of Interest) dimensions.

**Figure 18:**
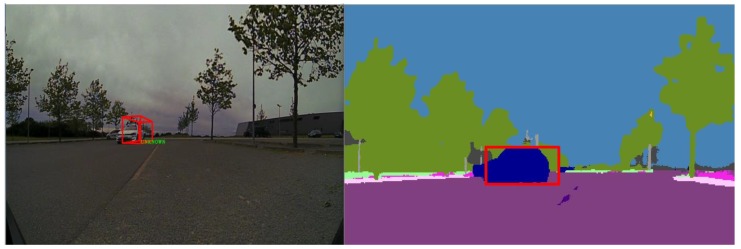
Example of mismatch in the validation procedure. On the left-hand side, we observe the projected fused object on the RGB image with the semantic class UNKNOWN. In the right image, the same object is projected onto the semantic class image in a region with the dominant class car. The class mismatch is represented using red color.

Finally, the dominant category is extracted by retrieving the class that corresponds to the index where the maximum membership value is stored. The class is extracted from the fused object in a similar manner, being the class corresponding to the index where the maximum value is stored. Lastly, a comparison between the two found classes is performed to see if there is a match. For evaluation purposes, we draw a green square in the semantic segmentation image if the two classes match and a red square if there is no match, as depicted in Figure 18.

**Figure 19:**
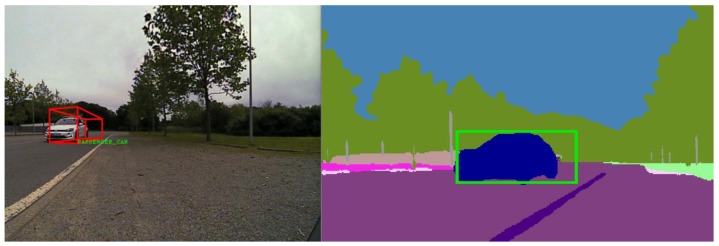
Example of successful validation. In the left image, the super-sensor object is projected onto the RGB image. In the right image, the same object is projected onto the semantic segmentation image. The semantic classes’ match is represented using green color.

In Figure 18, the trifocal camera was unable to detect the correct class of the vehicle, and it has labeled it as unknown. In the segmented image, the dominant region detected is “car”, so the two classes do not match, hence we draw a red square. If the classes match, a green square is drawn over the region of interest, as seen in Figure 19.

For increasing the robustness of our solution, a double check is performed for each fusion object. This means that when performing the validation algorithm, we check both the UKF approach to fusion and the neural fusion. This is done because there are cases when, due to sensor errors, one of the fusion algorithms does not offer good results. However, when the two fusions are combined, the final validation decision is more robust and reliable. Both hypotheses from the fusion results are considered. If, when each hypothesis has a class identical to the one from the segmentation image, the result is labeled as a hit, then it is validated; otherwise, it is labeled as a miss and it is not validated.

## 4. Experimental Results

In this section, we evaluate the results of the proposed solution with respect to the position given by a high-precision GPS placed on a tracked target vehicle. The system on which we have tested our method has an Intel i7-4770 K CPU with 3.5 GHz frequency and 8 GB of RAM memory. This section is split into two subsections: Section 4.1 presents the characteristics of the sensors used, and Section 4.2 presents the results of our solutions in different scenarios.

### 4.1. Experimental Setup

The main characteristics of the GPS system that was used to obtain the information from a target vehicle are displayed in Table 1 [48]. We refer to the vehicle on which the GPS is mounted as the target vehicle and the car on which the sensors are mounted as the reference (or ego) vehicle. We select the nearest neighbor to the target vehicle in order to assess whether the position of the predicted cuboids is correctly found. In all of our experiments, the virtual image on which the objects are projected has the horizontal (*y*) axis reversed. The running time of the proposed solution is 90 ms, and the obtained average error is 0.8 m. The main characteristics of each sensor from the ego vehicle are detailed in Table 2, Table 3, Table 4, Table 5, Table 6 and Table 7 below.

Additionally, for the precise position of the ego vehicle, the GPS system measures the yaw (heading), pitch and roll angles, which define its complete attitude in the 3D space. These angles are frequently used, in three consecutive rotations (first heading, then pitch and finally roll), to transform a measurement from the ego coordinate frame into the navigation (Earth) coordinate frame and vice-versa. The navigation coordinate frame is the orientation on the Earth at the current location with axes of north, east and down. The direction of axes for zero heading, pitch and roll values are defined in Table 1. If $V_{e}$ is the vector measured in the ego coordinate frame and $V_{n}$ is the vector measured in the navigation coordinate frame, the two vectors are related by the heading angle (ψ), pitch angle (θ) and roll angle (ϕ) using Equation (40).

(40) $$V_{n}=\left(\begin{array}{ccc} \cos\left(\psi \right) & -\sin\left(\psi \right) & 0\\ \sin\left(\psi \right) & \cos\left(\psi \right) & 0\\ 0 & 0 & 1 \end{array}\right)\cdot \left(\begin{array}{ccc} \cos\left(\theta \right) & 0 & \sin\left(\theta \right)\\ 0 & 1 & 0\\ -\sin\left(\theta \right) & 0 & \cos\left(\theta \right) \end{array}\right)\cdot \left(\begin{array}{ccc} 1 & 0 & 0\\ 0 & \cos\left(\phi \right) & -\sin\left(\phi \right)\\ 0 & \sin\left(\phi \right) & \cos\left(\phi \right) \end{array}\right)\cdot V_{e}$$

The characteristics of the 16 L LIDAR used to detect the objects are illustrated in Table 2 [48] below, while the main characteristics of the 4 L LIDAR are presented in Table 3.

The main features of the used RADAR sensor are displayed in Table 4, while the features available for the trifocal camera are described in Table 5.

### 4.2. Experiments and Validation

For evaluating the trifocal data association algorithm, multiple scenarios were covered by the ego vehicle, equipped with the mentioned sensors, and the target vehicle, for example:

- the ego followed the target vehicle on multiple road scenarios (driving straight, taking curves and so on);
- the ego vehicle was followed by the target vehicle, and the target overtook the ego;
- the target vehicle approached the front of the ego vehicle in a different lane.

**Figure 20:**
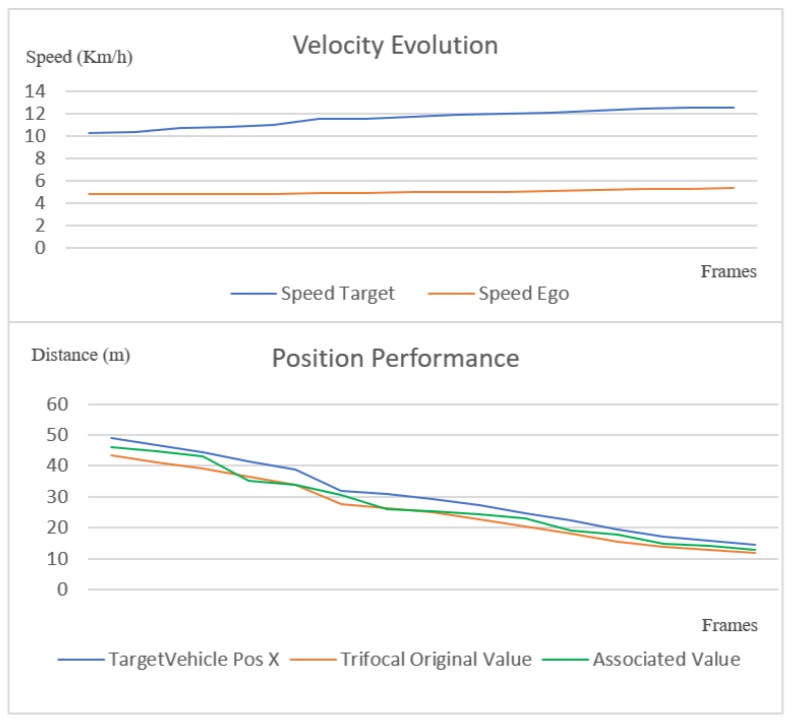
Speed chart (top) and position chart (bottom) of a trifocal object association scenario.

**Figure 21:**
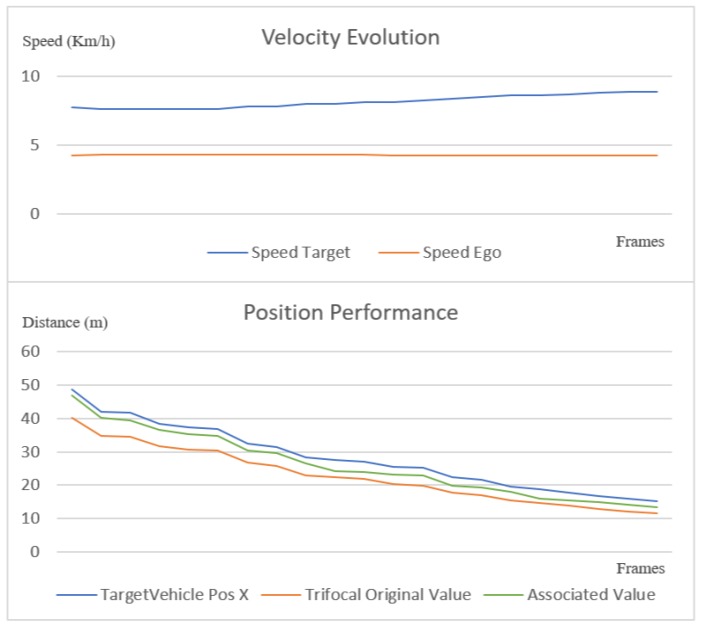
Another scenario that demonstrates the performance of the object association.

All covered scenarios were approached at different speeds of the target and ego vehicles, respectively. In the charts in Figure 20 and Figure 21, we illustrate the position of the trifocal object before and after the association with respect to the position of the target vehicle. A nearest neighbor association was performed between the target vehicle position and the enhanced trifocal object position to illustrate the accuracy of the trifocal data association process. The association accuracy is limited by the position accuracy of the object with whom the trifocal object is associated (the LIDAR object in this case). 

**Figure 22:**
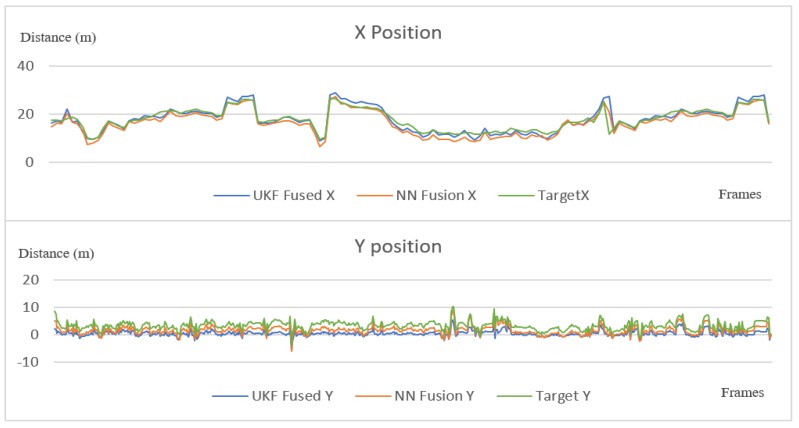
Position performance on the x and y axes of the proposed sensor fusion methods and the target vehicle.

In Figure 20, Figure 21 and Figure 22, on the horizontal *x* axis, we represent the number of frames on which we verified the association, and on the *y* axis, we represent the distance to the target vehicle. To make a better idea how the ego and target vehicles are moving relatively one to another, in the top pat of Figure 20 and Figure 21 the velocity evolution across the tested frames is displayed. The velocity chart has on the horizontal x axis the number of frames and on the vertical y axis the speed in Km/h. In the scenario presented in Figure 20, the target vehicle is accelerating while the ego vehicle is maintaining a fairly constant velocity. The green line represents the final position of the associated trifocal object that is closest to the target vehicle, depicted in blue color.

In Figure 21, another scenario is depicted where the target and ego vehicles at first move at similar speeds, and then the target vehicle starts to increase its speed. The position chart shows that the corrected vehicle position is closer to the ground truth than the original value.

The target tracking has been tailored for our specific input and objects. To evaluate the target tracking, we use two metrics: Multiple Object Tracking Precision (MOTP) and Multiple Object Tracking Accuracy (MOTA). MOTA combines true positives, true negatives and *ID* switch to indicate the overall performance of the tracker (41). By *t* we indicate the timestamp, and by *GT* we refer to the ground truth.

(41) $$\mathrm{MOTA}=1-\frac{\sum_{t}FN_{t}+FP_{t}+IDSW_{t}}{\sum_{t}GT_{t}}$$

The value of MOTA is maximized at 100, and it can also be negative if the number of errors exceeds the number of objects. The MOTP metric, from equation 42, on the other hand, refers to the averaged differences between true positives and ground truth. It gives the average overlap between the correctly identified tracks and the detected objects, as follows:

(42) $$\mathrm{MOTP}=\frac{\sum_{t,i}d_{t,i}}{\sum_{i}c_{t}}$$

where $c_{t}$ denotes the degree of tracker target match in frame *t*, and $d_{t,i}$ is the bounding box overlap between tracked target *i* and the ground truth object. More information about the tracking metrics is presented by Bernardin et al. in [49]. The scores of the evaluation and comparison with the global nearest neighbor method (GNN) are displayed in Table 6.

The dataset used for the evaluation in Table 6 contains real-world scenes covering different difficult scenarios in various weather conditions with ground truth data. The results from Table 6 indicate a relatively high degree of accuracy and precision for the tracker. The highest miss-rate, as we have observed, was for large objects, which across consecutive frames presented sporadic fluctuation with respect to their dimensions, semantic class and position. We also have to mention the fact that tracking 3D objects depends on the quality of the object segmentation. In Table 7, a comparison of the proposed tracking solution is done with other solutions available in the literature on the KITTI car dataset. The metrics used are MOTA and MOTP as well as running time. As mentioned before, the proposed association and tracking solution is able to track objects of multiple classes, not just cars.

The single-layer perceptron has been trained on 800 data points and evaluated on 465 points, obtaining an accuracy of 94% on the test set. For evaluating the sensor fusion, we have compared the position results from our fusion solution with the results from the ground truth given by the GPS. Some position estimates from different frames and scenarios, which were randomly selected, can be seen in Table 8. In Figure 22, we plot the results and ground truth values. The diagrams in Figure 22 have been plotted on a number of over 900 frames, where the ego vehicle is following the target vehicle in a scenario that includes straight driving, turns and driving at different speeds. As it can be seen, the two different types of fusion are very close to the target vehicle position, and follow the exact motion pattern of the target vehicle. 

The sensor errors also contribute to the small difference on the *x* and *y* axes seen between the ground truth and our result.

**Figure 23:**
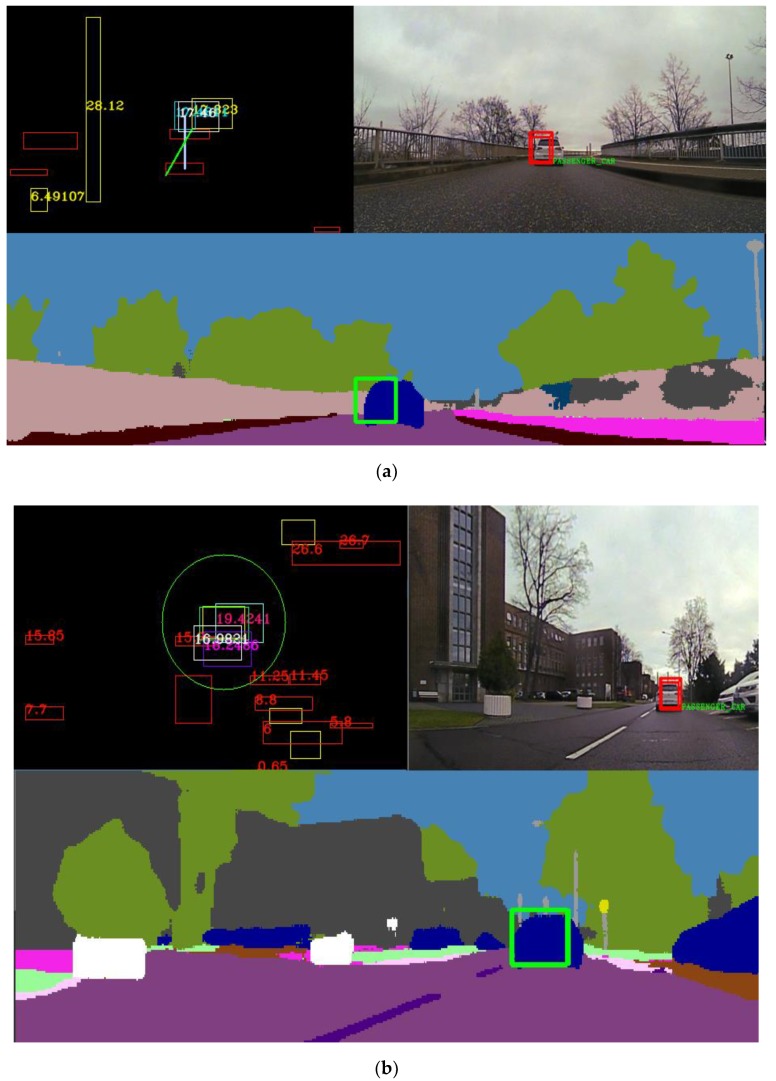
(**a**) In the top left picture, we illustrate the birds-eye view virtual image of the scene, where all objects are projected. In the top-right figure, we show the RGB image and the validated and stabilized super-sensor object projected on it. In the bottom image, the semantic segmentation image is depicted, and the validated super-sensor object is displayed with a green rectangle. In this scenario, only the UKF fusion was enabled. (**b**) The presented algorithm is depicted running in a heavy clutter scenario. The top-left figure is the birds-eye view virtual image. The top-right figure is the RGB image with the validated super-sensor object projected onto it. The bottom image displays the validated fused object projected onto the semantic segmentation image. In this scenario, both the UKF and neural fusion methods were enabled.

The trifocal data association, tracking and sensor fusion schemes help in stabilizing object parameters, chiefly among these, the object position. The validation of the sensor fusion results is achieved by comparing the semantic class of the fused object with the dominant semantic class from the region of interest corresponding to the projection of the fused object onto the semantic segmentation image. In Figure 23, two similar scenarios where a vehicle is followed in different environments are represented. In these scenarios, only the fused and validated objects are projected in the intensity image. In both cases, a vehicle is followed, and the redundant and complementary sensor information is used to determine the position, velocity and semantic class of this vehicle, among other parameters. In Figure 23a, only the UKF fusion was enabled and displayed. In Figure 23b, the same validation and stabilization algorithm is run with both fusion methods in heavy clutter. We can see cuboids from the trifocal camera, 16 L and 4 L LDAR that are mostly noise or small objects. The same color legend is used to represent the objects coming from different sensors as the one presented in Section 3.6.

**Figure 24:**
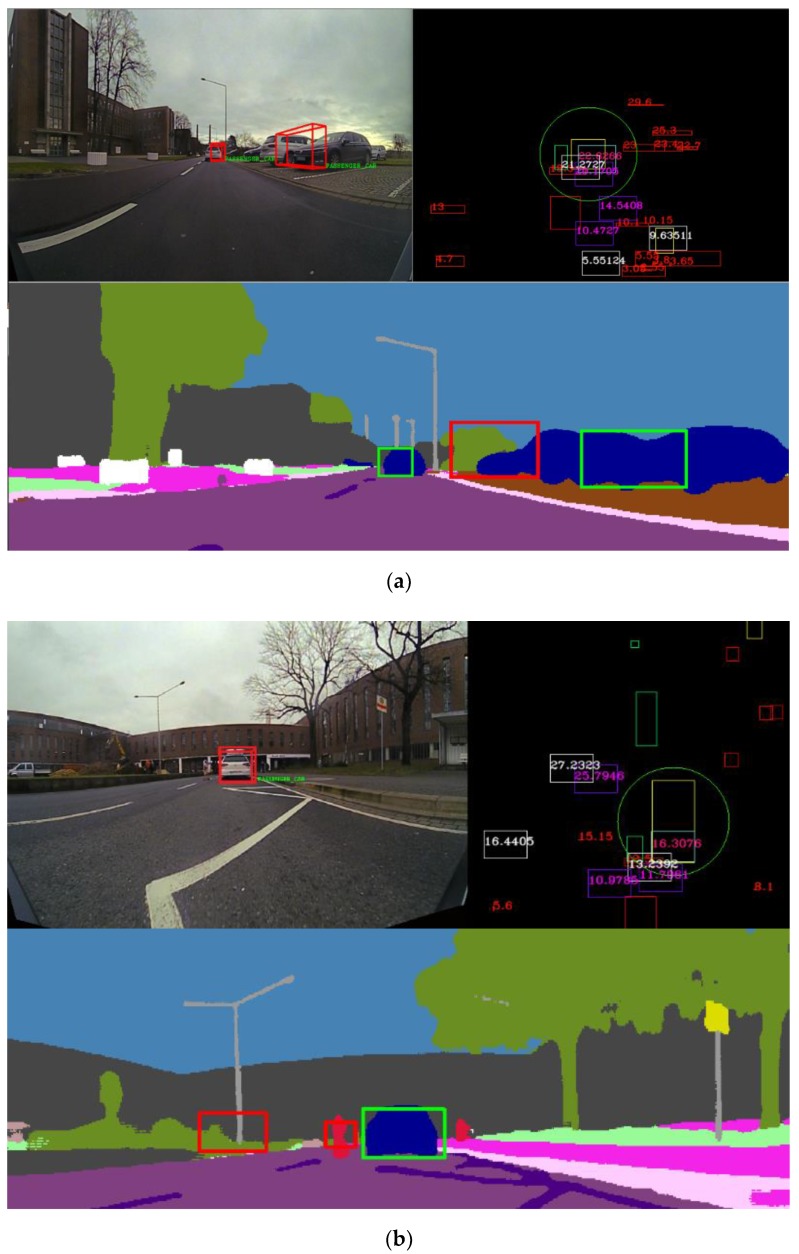
(**a**) In the top-right image, we observe the birds-eye view virtual scene of the real-world scenario, where all objects are projected. We note the presence of multiple super-sensor objects. In the bottom figure, the fused objects are projected onto the semantic segmentation image, and if the object is validated, a green rectangle is drawn; otherwise, the rectangle drawn has red color. In the top-left image, only the validated super-sensor objects are projected onto the RGB image. (**b**) Another scene where we note the presence of multiple super-sensor objects. In the top right, the birds-eye view image is depicted. The bottom image shows the semantic image with the validated super-sensor objects drawn in green and objects that are not validated drawn in red. The top-left image illustrates the RGB image, where the super-sensor objects are displayed.

In Figure 24, multiple fused objects are validated by projection onto the semantic segmentation image. In Figure 24a, two objects are validated, and for a third object there is a class mismatch. The two validated objects are projected onto the semantic segmentation image. In Figure 24b, we can observe a scenario where there are multiple fused objects that are not validated. The target vehicle has made a sudden slight right; even so, the data fused from multiple sensors are able to capture the position of the car and validate its semantic class.

## 5. Conclusions

In this paper, we have highlighted the difficulties that can appear in the field of autonomous driving when dealing with multi-sensor systems, and we have presented an original method for stabilizing and validating 3D object positions coming from several types of complementary sensors. We have proposed and implemented four contributions in various stages of the processing pipeline of the stabilization and validation processes. First of all, we have developed a two-step data association and tracking method that combines, in a weighted manner, motion and appearance features for 3D objects with different motion models, in order to improve the results of the data association and tracking of 3D objects provided by a 16 L LIDAR. Furthermore, we create a novel data association approach for finding the correct correspondences between trifocal camera objects and super-sensor objects, with the purpose of enriching the super-sensor information with the semantic class of the object. Moreover, we propose an object-level fusion architecture that combines a white box fusion method based on UKF with a black box fusion based on a single-layer perceptron, for stabilizing the position of redundant objects received from four types of complementary sensors: 4 L LIDAR, 16 L LIDAR, trifocal camera and RADAR.

The correctness of the position of the resultant fused 3D cuboids was verified using a semantic segmentation image obtained from an ERF neural network. Validated objects were displayed along with their class in the undistorted intensity image. The proposed solution has a real-time performance of 90 ms, and it was evaluated using real-world traffic data collected in different driving scenarios. The results of our solution were compared to ground truth data obtained from a high-precision GPS, which has a 2 cm-level accuracy, obtaining an average error of 0.8 m.

For future work, we plan to optimize the proposed approach using a GPU to reduce the running time as well as to incorporate map data to improve the validation process and eliminate false hypotheses.

## Figures and Tables

**Table 1:** GPS characteristics. The column numbers represent the following: I. Standard, II. Positioning, III. Position Accuracy, IV. Velocity Accuracy, V. Roll/pitch Accuracy (1σ), VI. Heading Accuracy (1σ)^2^, VII. Track angle Accuracy (1σ)^3^, VIII. Slip Angle Accuracy (1σ)^4^ IX. Axis, X. Earth Axis (Direction), XI. Ego Vehicle Axis (Direction).

I	II	III	IV	V	VI	VII	VIII	IX	X	XI
RT3003	L1, L2	0.01 m	0.05 Km/h	0.03°	0.1°	0.07°	0.15°	X, Y, Z	North, East, Down	Forward, Right, Down

**Table 2:** 16 L LIDAR characteristics.

Feature
Time of flight distance measurement with calibrated reflective
16 channels
Measurement range up to 100 m
Accuracy +/−3 cm
Dual returns
Field of view (vertical): 30° (+15° to −15°)
Angular resolution (vertical): 2°
Field of view (horizontal/azimuth): 360°
Angular resolution (horizontal/azimuth): 0.1°–0.4°
Rotation rate: 5–20 Hz

**Table 3:** 4 L LIDAR characteristics.

Feature
Time of flight distance measurement with calibrated reflective
4 channels
Measurement range up to 327 m
Accuracy +/−3 cm
Dual returns
Field of view (vertical): 3.2°
Angular resolution (vertical): 4 Layers @ 0.8°
Field of view (horizontal/azimuth): 145°
Angular resolution (horizontal/azimuth): 0.25°
Rotation rate: 12.5 Hz

**Table 4:** RADAR characteristics.

Feature
Operation Frequency 77 Ghz
Distance Measurement 0.25–250 m
Accuracy for distance measurement +/−2 m
Speed Measurement −400 + 200 kph
Sensitivity 0.1 kph

**Table 5:** Trifocal camera characteristics.

Feature
3 Cameras with FOV: 34, 46, 150 degrees
The sensor provides data for the following functions: Road infrastructure detection Object classification (cars, trucks, pedestrians etc.) 3D terrain perception (curb stones, generic road boundaries) Free space grid construction
Resolution: 1280 × 960, Bits per pixel 12
Upgrade Rate 33 ms
Color Grayscale

**Table 6:** Overall evaluation results.

Method	MOTA	MOTP	IDSW (Sum)	Running Time
Proposed	86.12%	91.01%	75	0.3 ms
GNN	31.88%	77.68%	511	0.05 ms

**Table 7:** Comparison with solutions from the KITTI benchmark.

No.	Solution Name	MOTA	MOTP	Running Time
1	Proposed Multi-Object Tracker	77.05%	81.65%	0.35 s
2	MDP [51]	76.59%	82.10%	0.9 s
3	CIWT [52]	75.39%	79.25%	0.3 s
4	DCO-X [53]	68.11%	78.85%	0.9 s

**Table 8:** Different fusion position samples.

Fused Object UKF		Fused Object NN		GPS Ground Truth	
*X* (*m*)	*Y* (*m*)	*X* (*m*)	*Y* (*m*)	*X* (*m*)	*Y* (*m*)
15.46	0.94	14.87	1.1	16.01	1.3
17.04	1.57	16.95	1.87	16.71	1.44
24.37	0.85	24.36	1.62	25.29	1.56
15.16	1.04	15.24	0.9	18.01	1.03
9.79	1.00	8.1	0.71	10.03	1.4
